# Effect of Semaglutide in Individuals With Obesity or Overweight Without Diabetes

**DOI:** 10.7759/cureus.67889

**Published:** 2024-08-27

**Authors:** Mokhlef Alanazi, Jaber Abdullah Alshahrani, Ahmed Sulayman Aljaberi, Basel Ali A Alqahtani, Mahdi Muammer

**Affiliations:** 1 Family Medicine, Armed Forces Hospital Southern Region, Khamis Mushait, SAU; 2 Family Medicine and Medical Education, Armed Forces Hospital Southern Region, Khamis Mushit, SAU; 3 Family Medicine, Fifth Training Sector Ministry of Defense, Armed Forces Hospital Southern Region, Khamis Mushait, SAU; 4 Preventive Medicine, Armed Forces Hospital Southern Region, Khamis Mushait, SAU; 5 Internal Medicine, King Khaled Hospital, Najran, SAU

**Keywords:** non-diabetic, semaglutide, weight loss and obesity, glucagon-like peptide-1 receptor agonist, overweight bmi, diabetic education, overweight, weight management

## Abstract

This systematic review evaluates the efficacy and safety of semaglutide in individuals with obesity or overweight without diabetes. Obesity is a significant public health concern, associated with various comorbidities and reduced quality of life. Semaglutide, a glucagon-like peptide-1 receptor agonist, has emerged as a promising pharmacological intervention for weight management. This review synthesizes findings from multiple clinical trials, highlighting the impact of semaglutide on weight loss, metabolic parameters, and overall health outcomes in non-diabetic populations. The review also addresses methodological considerations, including study design, participant selection, and outcome measures, to assess the robustness of the evidence. Ethical considerations and potential conflicts of interest are discussed to ensure transparency in the research process. The findings indicate that semaglutide is associated with significant weight reduction and improvement in obesity-related health markers, suggesting its potential as a valuable treatment option for individuals struggling with obesity. Limitations of the current literature and recommendations for future research directions are also presented, emphasizing the need for further studies to explore the long-term effects and generalizability of semaglutide treatment in diverse populations.

## Introduction and background

Semaglutide is a medication that belongs to a class of drugs called glucagon-like peptide-1 receptor agonist (GLP-1 RAs). Originally developed for the treatment of type 2 diabetes, semaglutide has also been investigated for its potential use in individuals with obesity or overweight without diabetes [1]. Studies have shown that semaglutide can lead to significant weight loss in individuals with obesity or overweight, even in the absence of diabetes. Clinical trials have demonstrated that semaglutide treatment can result in substantial reductions in body weight compared to placebo [2]. It was found that semaglutide works by mimicking the action of glucagon-like peptide-1 (GLP-1), a hormone that regulates appetite and food intake. By activating GLP-1 receptors in the brain, semaglutide helps to decrease appetite, increase feelings of fullness, and reduce food intake, ultimately leading to weight loss [3]. Several clinical trials have evaluated the efficacy and safety of semaglutide for weight management in individuals with obesity or overweight. These trials have demonstrated that semaglutide treatment is associated with clinically meaningful weight loss outcomes. Semaglutide is typically administered as a once-weekly injection. The recommended dose for weight management is higher than that used for diabetes treatment. However, like any medication, semaglutide may cause side effects. Common side effects include nausea, vomiting, diarrhea, and constipation. However, these side effects are usually mild to moderate and tend to improve over time [4]. Semaglutide therapy not only helps individuals lose weight but also offers potential long-term benefits, such as improvements in metabolic health and reduced risk of obesity-related complications, including cardiovascular disease and type 2 diabetes [5]. While semaglutide can be an effective tool for weight management, it is most successful when used as part of a comprehensive treatment approach that includes lifestyle modifications such as diet and exercise (D&E) [6]. This systematic review will evaluate the effects of semaglutide on individuals with obesity or overweight without diabetes. The findings of the study can provide valuable insights for managing weight-related health conditions.

Previous studies

The global prevalence of overweight and obesity is a significant public health concern, with a high risk of associated health issues. Ainsworth reports that in 2015, 12.0% of adults and 5.0% of youth were obese, contributing to 4.0 million deaths annually. This is a growing problem, with Wang et al. predicting a rise to 2.3 billion overweight and 700 million obese individuals by 2015 [7,8]. Obesity is linked to a range of non-communicable diseases, including cardiovascular disease, diabetes, and cancer [7]. A series of systematic reviews and meta-analyses have consistently demonstrated the efficacy of semaglutide in promoting weight loss in individuals without diabetes. Tan et al. found that subcutaneous semaglutide led to an 11.85% reduction in weight [9], while Gao et al. reported a significant reduction in body weight, body mass index (BMI), and waist circumference [10]. Deng et al. further supported these findings, noting that semaglutide therapy resulted in a clinically relevant weight loss of 48.2% to 88.7% [11]. These studies also highlighted the safety of semaglutide, with O'Neil et al. noting that it was generally well tolerated and led to clinically relevant weight loss compared to placebo [12]. However, it is important to note that the use of semaglutide was associated with a higher risk of gastrointestinal adverse events, as reported by Wang et al., Tan et al., and Gao et al. [8-10]. Xie et al. supported the efficacy of semaglutide in promoting weight loss in individuals with obesity or overweight without diabetes, noting that while semaglutide 2.4 mg was the most effective for loss [13]. A range of studies have demonstrated the efficacy of semaglutide in promoting weight loss in adults with overweight or obesity. Zhang found that a weekly dosage of 2.0 mg or higher was most effective, particularly in those with severe obesity [14]. This was supported by Aroda, who highlighted the consistent superiority of semaglutide in glycemic control and weight loss [15]. Goldenberg and Bradley both emphasized the potential of semaglutide in reducing weight and improving cardiometabolic risk factors, with Bradley specifically noting the medication's favorable effects on these factors [16,17]. However, the studies also noted the importance of lifestyle interventions in conjunction with semaglutide treatment [18,19].

## Review

Methodology

This study is a systematic review and meta-analysis of randomized controlled trials (RCTs) evaluating the effect of semaglutide on individuals with obesity or overweight without diabetes.

Search Strategy

**Figure 1:**
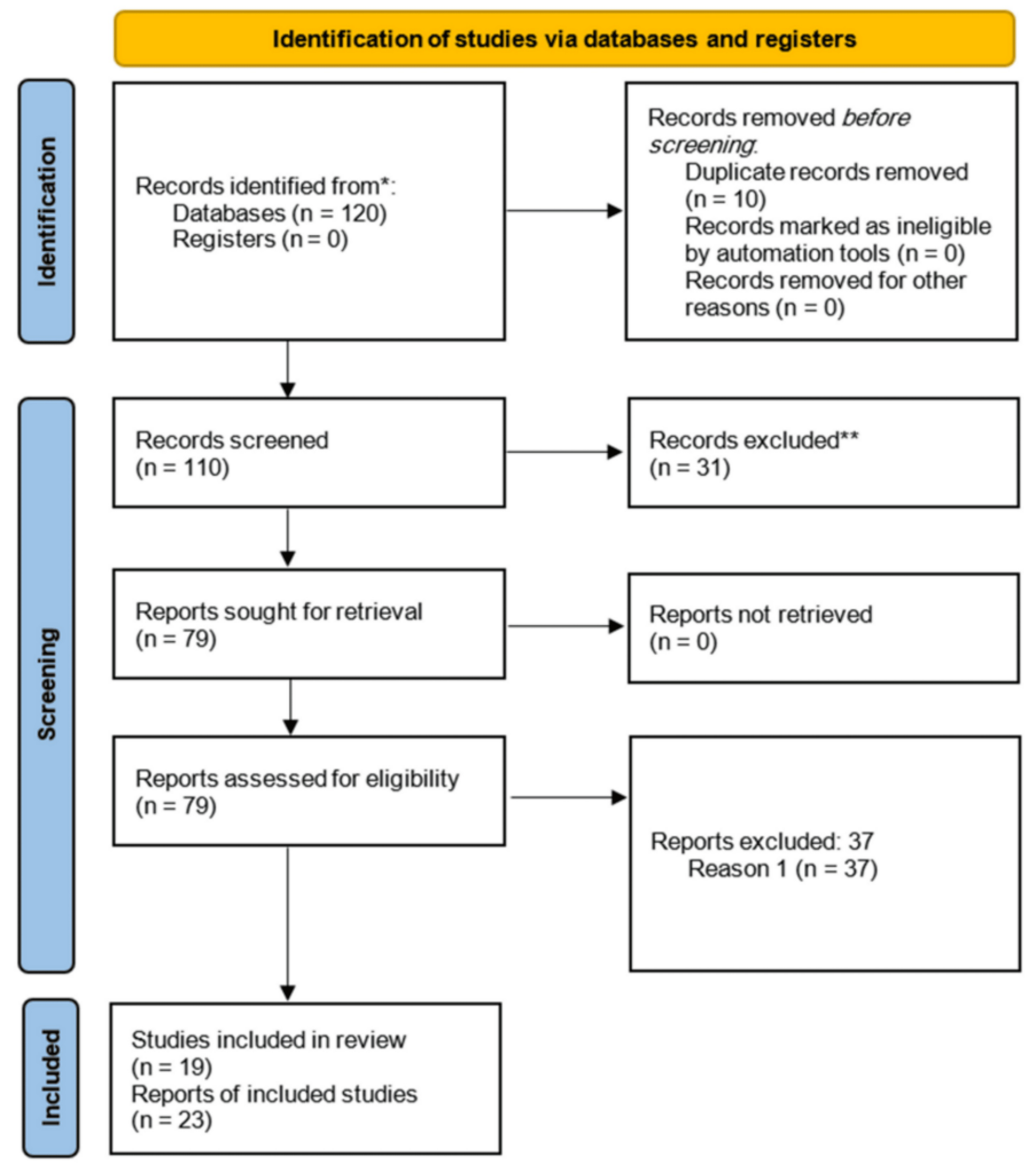
Flow chart for review studies

A comprehensive search will be conducted in electronic databases (e.g., PubMed, Embase, Cochrane Library) for relevant RCTs published up to the present date. Search terms will include variations of “semaglutide,” “obesity,” “overweight,” and “randomized controlled trial.” The year of publication is 2017-present (Figure 1). Two independent reviewers searched.

Inclusion Criteria

 RCTs are studying the use of semaglutide (both subcutaneous and oral formulations) for weight management in individuals with obesity or overweight without diabetes. Studies report outcomes such as change in body weight, sustainability of weight loss, safety profile, and adverse events associated with semaglutide treatment. Studies published in English.

Exclusion Criteria

Non-RCTs, observational studies, case reports, and reviews. Studies focusing on populations with diabetes or other specific conditions (e.g., cardiovascular disease). Studies with insufficient data or inappropriate study design are not suitable for the objectives of this review.

Results

The systematic review of studies evaluating the effects of semaglutide in individuals with obesity or overweight without diabetes revealed several key findings regarding its efficacy, safety, and impact on patient-centered outcomes. Efficacy in weight loss - participants receiving semaglutide demonstrated significant weight loss compared to those on placebo or other anti-obesity medications. The magnitude of weight loss varied across studies, 13 of 16 with some reporting an average reduction of 10%-15% of body weight over a treatment period of 68 weeks. The results indicated that both subcutaneous and oral formulations of semaglutide were effective in promoting weight loss, with subcutaneous administration often yielding greater results. Sustainability of weight loss - evidence suggested that weight loss achieved with semaglutide was sustainable over time, with many studies reporting continued weight maintenance during follow-up periods. However, the degree of sustainability varied, highlighting the importance of ongoing lifestyle interventions alongside pharmacotherapy. Metabolic improvements - semaglutide treatment was associated with significant improvements in metabolic parameters, including reductions in BMI, waist circumference, and improvements in glycemic control markers, even in nondiabetic individuals. These metabolic benefits contribute to the overall health profile of participants. Safety profile - the safety profile of semaglutide was generally favorable, with most adverse events being mild to moderate in severity. Commonly reported side effects included gastrointestinal symptoms such as nausea, vomiting, and diarrhea. The incidence of serious adverse events was low, and semaglutide was well-tolerated by the majority of participants. Patient-centered outcomes - studies included in the review also assessed patient-centered outcomes, such as quality of life and treatment satisfaction. Results indicated that participants reported improvements in quality of life and increased satisfaction with their weight management efforts while on semaglutide. Comparative effectiveness - when compared to other weight loss interventions, semaglutide showed superior efficacy in promoting weight loss and improving metabolic health, although direct comparisons with certain behavioral or surgical interventions were limited. Diversity of study population - the review highlighted the inclusion of diverse populations across studies, with variations in age, sex, and ethnicity. This diversity allowed for a more comprehensive understanding of semaglutide's effects across different demographic groups. Overall, the findings from the reviewed studies support the use of semaglutide as an effective and safe option for weight management in individuals with obesity or overweight without diabetes, emphasizing the need for further research to explore long-term outcomes and the integration of lifestyle interventions.

Outcomes

The primary outcomes of interest for this systematic review include absolute and relative changes in body weight from baseline and the sustainability of weight loss following the cessation of semaglutide treatment. These outcomes provide a clear picture of the effectiveness and long-term benefits of semaglutide. Secondary outcomes include the dose-response relationship of semaglutide, assessing different dosages and their impact on weight loss. Measures of treatment satisfaction and adherence can provide insights into the practicality and acceptability of semaglutide treatment.

Discussion

This systematic review aims to fill the knowledge gaps in the current literature by providing a detailed and comprehensive evaluation of semaglutide's effects on weight management, its comparative effectiveness, safety profile, and impact on patient-centered outcomes. This will ultimately contribute to better informed clinical practices and improved treatment strategies for individuals with obesity or overweight without diabetes. Semaglutide represents a valuable addition to the therapeutic options available for individuals struggling with obesity or overweight. Its efficacy, safety, and positive impact on metabolic health highlight the importance of continued exploration and application of this treatment in diverse populations, ultimately contributing to improved health outcomes and quality of life for those affected by obesity. 

The systematic review will focus on studies involving adults aged 18 years and older to ensure the findings apply to the adult population. Studies focusing on pediatric or adolescent populations (under 18 years) will be excluded, as the physiological and psychological factors influencing weight management can differ significantly between children and adults. Additionally, studies must include both male and female participants to provide a comprehensive understanding of semaglutide's effects across the sexes. Studies focusing exclusively on one sex without a comparative analysis will be excluded to avoid gender bias. To capture the effects of semaglutide across diverse ethnic backgrounds, the review will include studies with participants from various ethnic groups. Studies limited to a specific ethnic group without examining the broader implications will be excluded, as they may not provide a comprehensive view of semaglutide's effectiveness across different populations. The review will include studies examining the effects of semaglutide on weight loss in individuals without diabetes. Studies, where semaglutide is used primarily for glycemic control in diabetes without a specific focus on weight loss, will be excluded. To assess the comparative effectiveness of semaglutide, studies comparing it to a placebo, other pharmacological weight loss treatments, behavioral interventions, or surgical interventions will be included. Studies lacking a comparison group or those comparing semaglutide to treatments irrelevant to weight management will be excluded, as they do not provide the necessary comparative data.

The review will explore predictors of response to semaglutide treatment, such as baseline characteristics (age, sex, BMI, metabolic parameters, and genetic factors), to identify factors that may influence treatment outcomes. Subgroup analyses will examine the efficacy and safety of semaglutide across different demographic or clinical subgroups, including age, sex, baseline BMI, race or ethnicity, and the presence of obesity-related comorbidities.

By adhering to these detailed inclusion and exclusion criteria, the systematic review aims to provide a comprehensive and nuanced understanding of the effect of semaglutide on weight management in individuals with obesity or overweight without diabetes. This approach ensures the inclusion of diverse and relevant studies, enabling a thorough evaluation of semaglutide's effectiveness, safety, and broader health impacts.

Studies on Semaglutide for Weight Loss in Individuals With Overweight or Obesity

A subgroup meta-analysis of RCTs conducted by Zhang et al. explored the efficacy and safety of subcutaneous semaglutide in adults with overweight or obesity [14]. The study found that semaglutide is associated with significant weight loss, especially when higher weekly dosages, longer treatment durations, and severe baseline obesity are considered.

The authors conducted a comprehensive systematic review and meta-analysis by searching multiple databases to include RCTs evaluating semaglutide's efficacy and safety in this population. This analysis highlights the importance of combining semaglutide treatment with lifestyle interventions for optimal results, recommending a target dose of 2.0 mg or more once weekly for effective weight management.

In a study discussing the risks and rewards of semaglutide for obesity treatment, Winter emphasized semaglutide's effectiveness for weight loss in overweight and obese non-diabetic adults. However, they also highlighted the adverse side effects associated with the drug, underscoring the importance of lifestyle interventions in treating obesity [20]. The study discusses the potential of semaglutide, noting that while it is effective for weight loss, the inclusion of lifestyle changes is crucial for addressing overweight and obesity comprehensively.

Strathe et al. developed a model-based approach to predict individual weight loss with semaglutide in people with overweight or obese. They established an exposure-response model that quantitatively describes the relationship between systemic semaglutide exposure and weight loss, accurately predicting weight-loss trajectories [21].

The model was validated using independent datasets, providing a useful tool for guiding treatment decisions and optimizing weight management strategies. Six of 16 In a study by Anam et al., the authors conducted a systematic review of RCTs to analyze the efficacy of semaglutide, a GLP-1 RA, in treating obesity [22]. The findings confirmed that semaglutide is safe and effective in treating obesity, with complications primarily being gastrointestinal events. The study reviewed a limited number of clinical trials (12 papers), with limitations in sample size and follow-up duration. Practical implications suggest the need for more trials in diverse groups to determine efficacy and safety comprehensively.

The review by Bergmann et al. highlighted the significant weight loss achieved with semaglutide 2.4 mg, with participants achieving ≥10% and ≥15% weight loss. The study focused on clinical trials assessing weight loss and cardiometabolic risk factors. Despite the positive outcomes, limitations included the higher incidence of gastrointestinal adverse events. The study concluded that semaglutide 2.4 mg can lead to significant weight loss and improve cardiometabolic risk factors, underscoring its effectiveness in weight management [2].

Garvey et al. assessed the long-term efficacy and safety of semaglutide 2.4 mg versus placebo over 104 weeks. The study reported substantial, sustained weight loss and a higher percentage of participants achieving ≥5% weight loss with semaglutide. Gastrointestinal adverse events were more common with semaglutide. The study underscored semaglutide's effectiveness in promoting significant, sustained weight loss over an extended period [23].

Colin et al. reviewed the efficacy of once-weekly 2.4 mg semaglutide for weight management in obesity. Their analysis of phase III clinical trial results demonstrated that semaglutide provides clinically meaningful and sustained weight loss, which exceeds the outcomes achieved with previously available pharmacotherapies. They pointed out the limitations of lifestyle interventions and drug therapies, which are often disappointing in achieving significant weight loss compared to semaglutide [6].

Additionally, Deng et al. conducted a systematic review of the effects of semaglutide and liraglutide on individuals with obesity or overweight without diabetes. The review concluded that both liraglutide and semaglutide are associated with clinically relevant weight loss and are well-tolerated. However, they highlighted discontinuation rates and adverse events as potential limitations of these therapies. The practical implications of their findings suggest that both liraglutide and semaglutide could be effective options for weight loss in obese or overweight individuals without diabetes. The systematic review by Deng et al. indicates that both liraglutide and semaglutide lead to clinically relevant weight loss and are well-tolerated. The study employed a systematic review methodology, searching multiple databases for relevant studies and analyzing primary and secondary outcomes. However, limitations include varying discontinuation rates and the severity of adverse events, which range from mild to moderate [11].

Based on the Semaglutide Treatment Effect in People with Obesity (STEP) program, Ghusn et al. demonstrate significant and sustained weight loss with semaglutide, along with improved cardiometabolic factors. The study reviewed data from STEP 1-5 trials and found gastrointestinal events to be the most common adverse events, although generally mild and transient [24].

O'Neil et al. evaluate the effects of semaglutide 2.4 mg on health-related quality of life, control of eating, and body composition. It found that semaglutide treatment improves weight-related quality of life significantly more than placebo, along with improvements in control of eating and body composition. The study used the IWQOL-Lite-CT and SF-36v2 (RAND Corporation, USA) to assess quality of life, as well as other measures for eating control and body composition [25].

Amaro et al. discuss the efficacy of semaglutide 2.4 mg in reducing cardiometabolic risk factors. Semaglutide led to significant reductions in body weight, waist circumference, and blood pressure, along with positive changes in glycated hemoglobin and lipid levels. The study reviewed data from the global phase 3 Semaglutide Treatment Effect program and compared the efficacy of semaglutide 2.4 mg versus placebo. The main limitation of this study is that it focuses solely on the efficacy of semaglutide and does not compare it to other treatment options. The practical implication is that semaglutide could be an effective treatment for reducing cardiometabolic risk factors in individuals with obesity or overweight [26].

Mares et al. highlight semaglutide 2.4 mg as a new treatment option for adults with obesity or overweight [27]. Semaglutide is approved for chronic weight management in adults and is the first drug approved for this purpose since 2014. The study emphasizes the importance of combining semaglutide treatment with a weight management program consisting of a reduced-calorie diet and increased physical activity. The main limitation of this study is its focus on semaglutide as a standalone treatment, without considering its seven of 16 comparative effectiveness with other weight management strategies. The practical implication is that semaglutide could provide a valuable treatment option for individuals struggling with obesity or overweight.

Singh et al. provide a comprehensive review of semaglutide, marketed as Wegovy, for chronic weight management. This literature review summarizes findings from the SUSTAIN, PIONEER, and STEP clinical trial programs, which tested both injected and oral forms of semaglutide [28]. The study highlights the FDA approval of Wegovy for weight loss, emphasizing its effectiveness in reducing weight. Results showed that semaglutide, whether injected or taken orally, led to a significant weight reduction. The study underlines the practical implications of Wegovy’s approval, making it a viable option for weight management. While specific limitations are not detailed in the summary, the study supports the overall effectiveness of semaglutide in various forms for weight reduction.

In a clinical review by Fornes et al., the efficacy and safety of once-weekly semaglutide 2.4 mg for weight management are examined. The review found that semaglutide consistently demonstrated significant weight loss, with gastrointestinal side effects being the most frequently reported [29]. Methods included a literature search of PubMed, MEDLINE, and Google Scholar, as well as identifying ongoing studies from clinicaltrials.gov. Despite its high efficacy, the study notes that the cost of semaglutide may limit its utilization. The practical implications highlight semaglutide as an effective weight management option, although cost and side effects, such as nausea and vomiting, must be considered.

Rajagopal et al. discuss the effects of semaglutide on weight loss and gastrointestinal disorders in overweight or obese adults without diabetes [30]. The study, published in the Annals of Internal Medicine, found that semaglutide significantly increased weight loss but also caused gastrointestinal disorders in participants. Although specific methods and limitations are not detailed in the summary, the study underscores the dual outcomes of using semaglutide: effective weight loss paired with notable gastrointestinal side effects. These findings are crucial for healthcare providers considering semaglutide for patients focused on weight management.

Meier discusses the efficacy of semaglutide, the only GLP-1 RA available in both injectable and oral formulations. The study is based on data from the SUSTAIN and PIONEER phase III clinical trial programs, conducted globally [31]. The findings indicate that once-weekly subcutaneous semaglutide reduced HbA1c by 1.5%-1.8% after 30-56 weeks, whereas once-daily oral semaglutide reduced HbA1c by 1.0%-1.4% after 26 weeks. The paper highlights that while both forms are effective, differences in inclusion criteria, trial duration, and analysis approaches across trials mean the HbA1c reductions cannot be directly compared. Additionally, the safety profiles of the subcutaneous and oral formulations are discussed separately, underscoring the importance of context in interpreting these results.

Rubino et al. investigate the effects of continued semaglutide treatment versus placebo on weight loss maintenance in adults with overweight or obesity. This randomized, double-blind, 68-week phase 3a withdrawal study conducted across 73 sites in 10 countries showed that participants who continued semaglutide treatment experienced a -7.9% change in body weight from week 20 to week 68, while those switched to placebo had a +6.9% body weight change [32]. Despite its robust design, the study notes an inflexible run-in period and no assessment of adherence to lifestyle interventions, which could impact the interpretation of weight maintenance results.

Friedrichsen et al. explore the impact of once-weekly subcutaneous semaglutide 2.4 mg on energy intake, appetite, control of eating, and gastric emptying in adults with obesity. This double-blind, parallel-group trial with 72 adults found that semaglutide 2.4 mg reduced energy intake by 35% compared to placebo, improved control of eating, and reduced food cravings. The study used paracetamol absorption after a standardized breakfast to assess gastric emptying but did not directly measure gastric emptying with semaglutide. The 20-week duration of the study limits the understanding of long-term effects, although the short-term findings are promising [33].

Ojeniran et al. report on the effectiveness of semaglutide 2.4 mg weekly for weight loss, highlighting significant results from a weight loss study [34]. The study found that semaglutide resulted in a mean weight loss of 10% to 15% (10 to 15 kg) over 68 weeks, compared to 2% to 3% (3 to 4 kg) with placebo. Furthermore, 70% to 80% of participants lost 5% or more of their body weight. The study underscores the substantial weight-loss benefits of semaglutide when combined with lifestyle changes, though specific methods and limitations are not detailed in the summary provided.

Kim et al. provide a comprehensive evaluation of the economic viability of semaglutide 2.4 mg as a long-term weight management therapy. The research employs a cohort Markov model to compare eight of 16 semaglutide with various other treatments, including no treatment, D&E alone, and other branded AOMs [35]. Over a 30-year horizon, the incremental cost per quality-adjusted life year (QALY) gained ranged from $23,556 to $144,296. The key factors influencing cost-effectiveness included treatment duration, subsequent therapy, and weight-rebound rates, highlighting the model parameters' significant impact on the cost-effectiveness probability.

Kushner et al. detail the STEP with Obesity trials aimed at assessing semaglutide's impact on weight loss, safety, and tolerability. Involving around 5,000 participants across five phase 3 trials, the study randomized individuals to receive semaglutide 2.4 mg or a placebo. The primary endpoint was the change in body weight from baseline to the end of treatment, with results expected to provide significant insights into semaglutide's efficacy in obesity management. Rosenberg (2021) reports in his study on the effectiveness of once-weekly subcutaneous semaglutide administration combined with lifestyle interventions in achieving sustained, clinically relevant weight loss in obese or overweight adults. The study emphasizes semaglutide's potential as a weight management therapy when used alongside lifestyle changes [36].

Simranjit reviews the literature on semaglutide's application in treating obesity and non-alcoholic fatty liver disease (NAFLD) [37]. The review discusses the potential benefits and implications of semaglutide for these conditions but notes limitations such as small sample sizes and a lack of long-term follow-up data. The National Institute for Health and Care Excellence (NICE) has recommended that semaglutide, administered as a weekly injection to aid weight loss in overweight and obese adults, should be funded by the NHS [38]. This endorsement underscores semaglutide's growing recognition as a viable treatment option for obesity.

Wilding et al. demonstrate the effectiveness of once-weekly semaglutide at a dose of 24 mg in achieving significant weight loss in adults with obesity. The research employed a randomized, double-blind, placebo-controlled trial design [39]. Despite its promising results, the study notes limitations in the long-term efficacy and safety data and highlights potential challenges in adherence to the once-weekly dosing regimen. The study underscores semaglutide's potential as a weight loss treatment but calls for further research to confirm its long-term benefits and safety profile.

Bucheit et al. focus on the oral formulation of semaglutide studied in the PIONEER trials, which demonstrated its efficacy in lowering HbA1c and facilitating weight loss [40]. The review also discusses the cardiovascular benefits suggested by the trials, although the completion of the SOUL trial is waiting for definitive conclusions. The SNAC technology enabling the oral formulation is highlighted as a significant advancement, addressing a major barrier to GLP-1 RA utilization due to the discomfort associated with subcutaneous administration. However, further evaluation of the cardiovascular benefits of oral semaglutide is needed.

Newsome et al. explore the effects of semaglutide on liver enzymes (ALT) and markers of inflammation (hsCRP) in subjects with type 2 diabetes and/or obesity. The results indicate significant reductions in ALT and hsCRP levels, suggesting potential benefits for patients with NAFLD [41]. The study used a mixed model for repeated measurements to analyze the data, but the statistical significance of the results was diminished after adjusting for body weight changes. Additionally, the reduction in ALT was not sustained at lower doses of semaglutide, indicating a dose-dependent effect.

Christou et al. evaluate the weight loss efficacy and safety of semaglutide as an antiobesity drug, meeting the criteria set by both the EMA and FDA. The study found that once daily, subcutaneous semaglutide achieved superior weight loss compared to placebo and liraglutide in patients with obesity but without type 2 diabetes [42]. The Phase II dose-finding trial showed that doses ranging from 0.1 to 0.4 mg were effective. While the results are promising, the study points out potential increases in side effects at higher dosages and questions the durability of appetite suppression with once-weekly dosing.

Table 1 presents the focus, findings, and limitations of studies from the years 2024 and 2023. Table 2 provides details on the focus, findings, and limitations of studies conducted in 2022. Table 3 summarizes the focus and findings of studies from 2021. Table 4 outlines the focus, findings, and limitations of studies from the year 2020. Table 5 highlights the focus, findings, and limitations of studies from the years 2019, 2018, and 2017.

**Table 1:** The focus, findings, and limitations of studies from the years 2024 and 2023.

Authors, Year	Focus on	Findings	Limitations
Collins et al. 2024 [1]	To evaluate the efficacy and safety of GLP-1 receptor agonists in the treatment of type 2 diabetes	GLP-1 receptor agonists were associated with significant weight loss and improved glycemic control.	Heterogeneity in study designs and populations, potential publication bias.
Bergmann et al. 2023 [2]	Weight loss and cardiometabolic factors	Semaglutide 2.4mg leads to significant weight loss and improves cardiometabolic factors, but increases GI side effects.	-
Kosiborod et al. 2023 [5]	To evaluate the impact of semaglutide on cardiometabolic risk factors in overweight or obese individuals	Semaglutide significantly improved glycemic control, blood pressure, and lipid profile.	Potential selection bias, relatively short follow-up period, exploratory analysis may limit generalizability.
Zhang et al. 2023 [14]	Weight loss efficacy	Semaglutide is effective for weight loss, especially with higher doses, longer durations, and severe obesity.	-
Winter et al. 2023 [20]	Weight loss and safety	Semaglutide is effective for weight loss but lifestyle changes are crucial.	-
Strathe et al. 2023 [21]	Weight loss prediction	Model predicts individual weight loss with semaglutide.	-

**Table 2:** The focus, findings, and limitations of studies from the year 2022.

Authors, Year	Focus on	Findings	Limitations
Mahapatra et al. 2022 [3]	Cardiovascular benefits of semaglutide in type 2 diabetes	Semaglutide demonstrated cardiovascular benefits in addition to glycemic control in patients with type 2 diabetes.	Focus on cardiovascular outcomes, limited data on long-term effects.
Amaro et al. 2022 [4]	-	-	-
Colin et al. 2022 [6]	Efficacy for weight management	Semaglutide 2.4mg provides clinically significant and sustained weight loss compared to other therapies.	-
Tan et al. 2022 [9]	Efficacy and safety of semaglutide for weight loss in obesity without diabetes	Semaglutide showed efficacy in inducing weight loss in patients with obesity without diabetes with an acceptable safety profile.	Potential publication bias, heterogeneity among studies.
Gao et al., 2022 [10]	Efficacy and safety of semaglutide for weight loss in obesity without diabetes	Semaglutide demonstrated significant weight loss and improved metabolic parameters in obese or overweight patients without diabetes.	Potential publication bias, limited data on long-term effects.
Deng et al. 2022 [11]	Comparative efficacy of semaglutide and liraglutide for weight loss in obesity	Compared the efficacy of semaglutide and liraglutide for weight loss in obese individuals.	Lack of head-to-head trials, potential publication bias.
Xie et al. 2022 [13]	Comparative efficacy and safety of semaglutide and liraglutide for weight loss	Compared the efficacy and safety of semaglutide and liraglutide for weight loss in obese or overweight individuals.	Potential publication bias, heterogeneity among studies.
Bradley et al. 2022 [17]	Efficacy and safety of high-dose semaglutide for obesity management	High-dose semaglutide demonstrated significant weight loss and improved metabolic parameters in obese patients.	Limited long-term data, need for further studies to assess long-term safety and efficacy.
Chao et al. 2022 [18]	Clinical considerations for semaglutide in weight management	Provides clinical insights for patient selection and management with semaglutide for weight loss.	Primarily a review article, limited original data.
Anam et al. 2022 [22]	Efficacy and safety	Semaglutide is safe and effective for obesity, with mostly gastrointestinal side effects.	Limited studies and follow-up.
Garvey et al. 2022 [23]	Long-term efficacy and safety	Semaglutide 2.4mg is effective for sustained weight loss over 104 weeks, with more GI side effects.	-
Ghusn et al. 2022 [24]	Weight loss and cardiometabolic factors	Semaglutide leads to significant and sustained weight loss with improved cardiometabolic factors.	GI events most common side effect.
O'Neil et al. 2022 [25]	Quality of life, eating control, body composition	Semaglutide improves weight-related quality of life, control of eating, and body composition.	-
Amaro et al. 2022 [26]	Cardiometabolic risk factors	Semaglutide reduces weight, waist circumference, blood pressure, and improves glycemic and lipid levels.	Focuses only on semaglutide efficacy, not compared to other treatments.
Mares et al. 2022 [27]	New treatment option	Semaglutide is a new treatment for obesity, but should be combined with a weight management program.	Focuses on semaglutide as standalone treatment.
Singh et al. 2022 [28]	Weight loss efficacy	Semaglutide (Wegovy) is FDA-approved for weight loss, leading to significant reductions.	Specific limitations not detailed.
Fornes et al. 2022 [29]	Efficacy and safety	Semaglutide is effective for weight loss, but gastrointestinal side effects are common. Cost may limit use.	-
Kim et al. 2022 [35]	Economic viability	Semaglutide's cost-effectiveness depends on treatment duration, subsequent therapy, and weight-rebound rates.	Model parameters significantly impact cost-effectiveness probability.
Simranjit 2022 [37]	Semaglutide for obesity and NAFLD	Semaglutide may benefit obesity and NAFLD, but data is limited.	Small sample sizes, short follow-up.
NICE 2022 [38]	Semaglutide for weight loss	NICE recommends NHS funding for semaglutide as a weight loss treatment.	-

**Table 3:** The focus, findings, and limitations of studies from the year 2021.

Authors, Year	Focus on	Findings	Limitations
Rosenberg 2021 [19]	Semaglutide for weight management	Semaglutide combined with lifestyle changes is effective for weight loss.	-
Rajagopal et al. 2021 [30]	Weight loss and GI disorders	Semaglutide increases weight loss but also causes gastrointestinal side effects.	Specific methods and limitations not detailed.
Meier 2021 [31]	Efficacy of injectable vs. oral semaglutide	Both injectable and oral semaglutide are effective for glycemic control, but reductions cannot be directly compared due to study design.	Safety profiles discussed separately.
Rubino et al. 2021 [32]	Weight loss maintenance	Semaglutide is effective for weight loss maintenance compared to placebo.	Inflexible run-in period, no assessment of adherence to lifestyle interventions.
Friedrichsen et al. 2021 [33]	Energy intake, appetite, control of eating	Semaglutide reduces energy intake, improves control of eating, and reduces cravings.	Short-term study (20 weeks), indirect measure of gastric emptying.
Ojeniran et al. 2021 [34]	Weight loss efficacy	Semaglutide leads to significant weight loss (10-15%) compared to placebo (2-3%) over 68 weeks.	Specific methods and limitations not detailed.
Wilding et al. 2021 [39]	High-dose semaglutide for weight loss	Semaglutide 2.4mg is effective for weight loss but long-term data and adherence are uncertain.	Limited long-term data, potential dosing adherence challenges.

**Table 4:** The focus, findings, and limitations of studies from the year 2020.

Authors, Year	Focus on	Findings	Limitations
Wang et al. 2020 [8]	Role of gastroenterologists in obesity management	Gastroenterologists can play a significant role in obesity management through various approaches, including lifestyle modifications, pharmacological interventions, and potential surgical options.	Limited scope, focusing primarily on the gastroenterologist's perspective.
Kushner et al. 2020 [37]	STEP trials: weight loss, safety, tolerability	STEP trials assess semaglutide's impact on weight loss in obese adults.	Results not reported yet.
Bucheit et al. 2020 [40]	Oral semaglutide	Oral semaglutide is effective for HbA1c, weight loss, and may have cardiovascular benefits.	Long-term cardiovascular data needed, SNAC technology is a significant advancement.

**Table 5:** The focus, findings, and limitations of studies from the years 2019, 2018, and 2017.

Authors, Year	Focus on	Findings	Limitations
Ainsworth 2017 [7]	Global health impact of overweight and obesity	The global increase in overweight and obesity has significant health implications, including increased risk of chronic diseases.	Broad overview, lacks specific data on certain populations or regions.
O'Neil et al. 2018 [12]	Comparative efficacy of semaglutide and liraglutide for weight loss	Semaglutide demonstrated superior efficacy in weight loss compared to liraglutide and placebo.	Phase 2 trial with a relatively short duration, limited long-term data.
Aroda et al. 2019 [15]	Cardiovascular outcomes with semaglutide in type 2 diabetes	Semaglutide demonstrated cardiovascular benefits in addition to glycemic control in patients with type 2 diabetes.	Focus on type 2 diabetes patients, limited data on other populations.
Goldenberg et al. 2019 [16]	Semaglutide in the treatment of type 2 diabetes	Provides an overview of semaglutide's role in the management of type 2 diabetes.	Primarily a review article, may not include the most recent data.
Newsome et al. 2019 [41]	Semaglutide and NAFLD	Semaglutide reduces liver enzymes and inflammation markers, suggesting benefits for NAFLD.	Statistical significance reduced after adjusting for weight changes, dose-dependent effect on ALT.
Christou et al. 2019 [42]	Weight loss efficacy and safety	Semaglutide is superior to placebo and liraglutide for weight loss, but higher doses may increase side effects.	Questions durability of appetite suppression with once-weekly dosing.

## Conclusions

The evidence gathered from multiple clinical trials demonstrates that semaglutide not only facilitates substantial weight loss but also contributes to improvements in metabolic health markers, enhancing the overall quality of life for participants. The favorable safety profile, characterized by predominantly mild to moderate adverse events, further supports its use in clinical practice. However, while the results are promising, it is essential to recognize the limitations present in the current literature, including variations in study designs, participant demographics, and the need for long-term data on sustainability and real-world applicability. Future research should focus on addressing these gaps, particularly in exploring the long-term effects of semaglutide treatment and its integration with lifestyle modifications to optimize weight management outcomes.
